# The first statewide, open access dataset tracking public records requests in New Jersey

**DOI:** 10.1016/j.dib.2020.106265

**Published:** 2020-09-02

**Authors:** Gavin C. Rozzi

**Affiliations:** a School of Natural Sciences and Mathematics, Stockton University, United States; b Rutgers Urban & Civic Informatics Lab, Edward J. Bloustein School of Planning & Public Policy, Rutgers, The State University of New Jersey, 33 Livingston Ave., Room 245 New Brunswick, NJ 08901, United States

**Keywords:** Freedom of information, Open data, Open public records, OPRA, FOIA, Government transparency, Open government, Information and communication technology

## Abstract

State freedom of information laws are vital mechanisms for providing public access to government records and supporting civic engagement through the effectuation of a public policy of transparency at the state level within the United States, not unlike their federal counterpart, the Freedom of Information Act (FOIA). New Jersey state law facilitates public access to government records under the Open Public Records Act (OPRA). Codified at N.J.S.A. 47:1A-1 et seq., OPRA applies to state, county and local public authorities but exempts the judicial and legislative branches from its disclosure requirements. Since OPRA took effect in 2002, it has been difficult to track the full extent of law's impact across New Jersey's 21 counties, 565 municipalities, and numerous state agencies, school districts and independent authorities, all of which must individually respond to requests under the law. To the best of the author's knowledge, no official source has compiled detailed metadata tracking the content and disposition of OPRA requests at the state, regional and municipal levels within New Jersey using individual requests, and authorities rarely proactively disclose their responses to requests they receive, necessitating further data collection to support research into the impacts of this law. This article presents the OPRAmachine dataset: data containing detailed metadata on public records requests submitted to state & local public authorities in New Jersey since October 2017 collected through the implementation of information and communication technologies (ICT) to facilitate the freedom of information request process. The data was collected using an open-source web interface that allowed users to submit an OPRA request to public authorities, with responses stored in a database and made available via the internet. After their request received a response, users were asked to answer a single survey question describing the status of their request, with their answer used to classify the request. Descriptive statistics, tables and frequencies were produced for the dataset and are included in this article. These data will assist state policymakers and other interested parties with assessing trends in OPRA requests across multiple types of public authorities & geographic regions. These data can inform more efficient government records management procedures, foster civic engagement by increasing government transparency and can inform the development of possible reforms to the OPRA law by showing trends in requests & responses that can be used to evaluate the law's implementation throughout the state.

## Specifications Table

**Table:** 

Subject	Law
Specific subject area	Freedom of information, public policy, government transparency
Type of data	CSV JSON RSS Table Figure
How data were acquired	Individuals were permitted to submit public records requests to public authorities in New Jersey under the Open Public Records Act (OPRA) using the Alaveteli [1] platform. Their requests were sent as emails to public authorities upon creation using a uniquely generated email address and responses from public authorities were recorded. Users were asked to answer a single multiple-choice question to describe the outcome of their request and their response was used to classify the status of the request.
Data format	Raw and analyzed
Parameters for data collection	Users were required to acknowledge that the content of their requests may be published and public authorities were notified that their responses will be published.
Description of data collection	Metadata for public records requests was created using the Alaveteli platform [1].
Data source location	New Jersey, USA
Data accessibility	Repository name: Mendeley Data Data identification number: 10.17632/bg8w9mfths.1 Direct URL to data: https://data.mendeley.com/datasets/bg8w9mfths A JSON REST API endpoint for recently filed requests is available at: https://opramachine.com/feed/search/%20(variety:sent%20OR%20variety:followup_sent%20OR%20variety:response%20OR%20variety:comment).json Additional API endpoints are described at: https://opramachine.com/help/api

## Value of the Data

• These data provide new insights regarding the volume, content and geographic distribution of public records requests & responses in New Jersey and can inform the ongoing policy debate regarding potential reforms to the state's freedom of information law, OPRA. This dataset offers detailed metadata for records requests submitted to public authorities within the state since 2017 that are suitable for evaluating trends in the law's implementation at the state, county and municipal levels.
• Policymakers, researchers, journalists, advocacy groups, and citizens can use the dataset to evaluate the OPRA law's implementation across multiple layers of government and regions in New Jersey. These data can support further research regarding how effective the law has been at accomplishing its public policy objectives of encouraging access to government records, as well as the extent to which individuals are requesting various types of records under OPRA.
• The dataset can be used to assess how well public agencies comply with OPRA's statutory 7 business day timeframe for responses to most requests, as response times are tracked for each request and average response times are also calculated for each county using aggregated request data.
• These data can assist public agencies in complying with the law by identifying trends in the most frequently requested records to inform the development of more efficient records management and archival procedures for government records, as well as to improve response times and reduce the administrative burden of complying with the law by highlighting frequently requested records and patterns in requester behavior. The burden of responding to voluminous requests is often cited as a concern by municipalities and other public authorities [2].
• This dataset provides a basis for a case study on a successful third-party eParticipation initiative deployed in New Jersey, as a traditional civic process – the submission of public records requests to state & local government – has been mediated by ICT [3] in order to produce the data described in this article and track the outcomes of requests.
• Amid the COVID-19 pandemic, these data can be used as a basis for further research on the extent to which public authorities in New Jersey have delayed responses to records requests as a consequence of the unique circumstances presented by COVID-19, as many employees have been working remotely and have limited access to archived government records. In one case, a consortium of journalists used a subset of this dataset to identify specific instances where municipal governments cited the pandemic as a basis to delay responses to public records requests beginning in March 2020 [4].

## 1 Data Description

### 1.1 OPRA request metadata

**Fig. 1:**
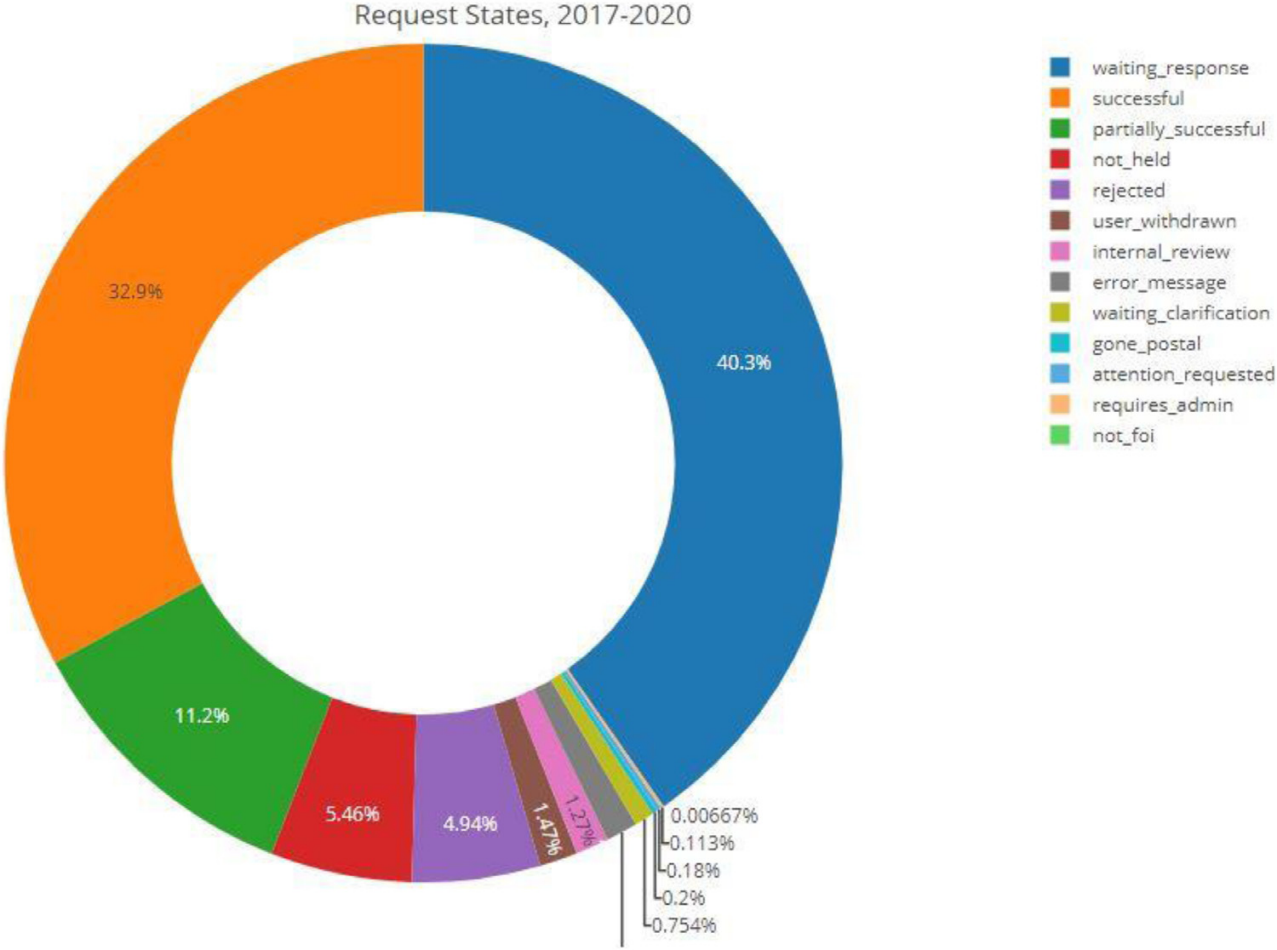
Percentages of request states based upon user classification Fig. 1 Distribution of OPRA request states as classified by OPRAmachine.com users from 2017-2020. The correlation between request states and survey responses in shown in Table 3.

The primary data file consists of a CSV (“request_data.csv”) containing metadata collected for all OPRA requests that have been submitted using the OPRAmachine.com web service since 2017. Each row represents data collected for a single public records request submitted by requesters [5]. Table 1 describes the content of each column of the main public records request dataset, while Fig. 1 shows the distribution of values for the described_state column for all requests represented in this data file. The value of described_state correlated with a response to a survey question presented to requesters after their request received a response from a public authority and was used to classify each request's status.

**Table 1:** Description of metadata collected for each public records request.

Column name	Column description	Data type
title	The summary of the contents of the user's public records request, as entered by the user	Character
url_title	The full URL to access the public records request on the web interface	Character
requested_by	The name of the individual that made the records request	Character
public_body_name	The name of the public authority that received the request.	Character
Info_request_batch_id	The ID of the batch, if this request was sent as a batch of identical request to multiple public authorities	Integer
described_state	The state the request has been classified as a result of survey response	Character
request_created_at	Timestamp of the request's creation by user	Timestamp
request_updated_at	Timestamp of when user last classified the request	Timestamp
date_initial_request_last_sent_at	Timestamp marking when request was last delivered to public authority	Timestamp
date_response_required_by	7 working days (minus legal holidays) from date_initial_request_last_sent_at	Timestamp
date_very_overdue_after	20 working days (minus holidays) from date_initial_request_last_sent_at	Timestamp
last_public_response_at	Timestamp marking when the last response was received from a public authority	Timestamp
tag_string	The tags used to categorize the public authority that this request was made to	Character
days_until_response	Difference in days between date_initial_request_last_sent_at and last_public_response_at	Float

### 1.2 County-level summaries

**Fig. 2:**
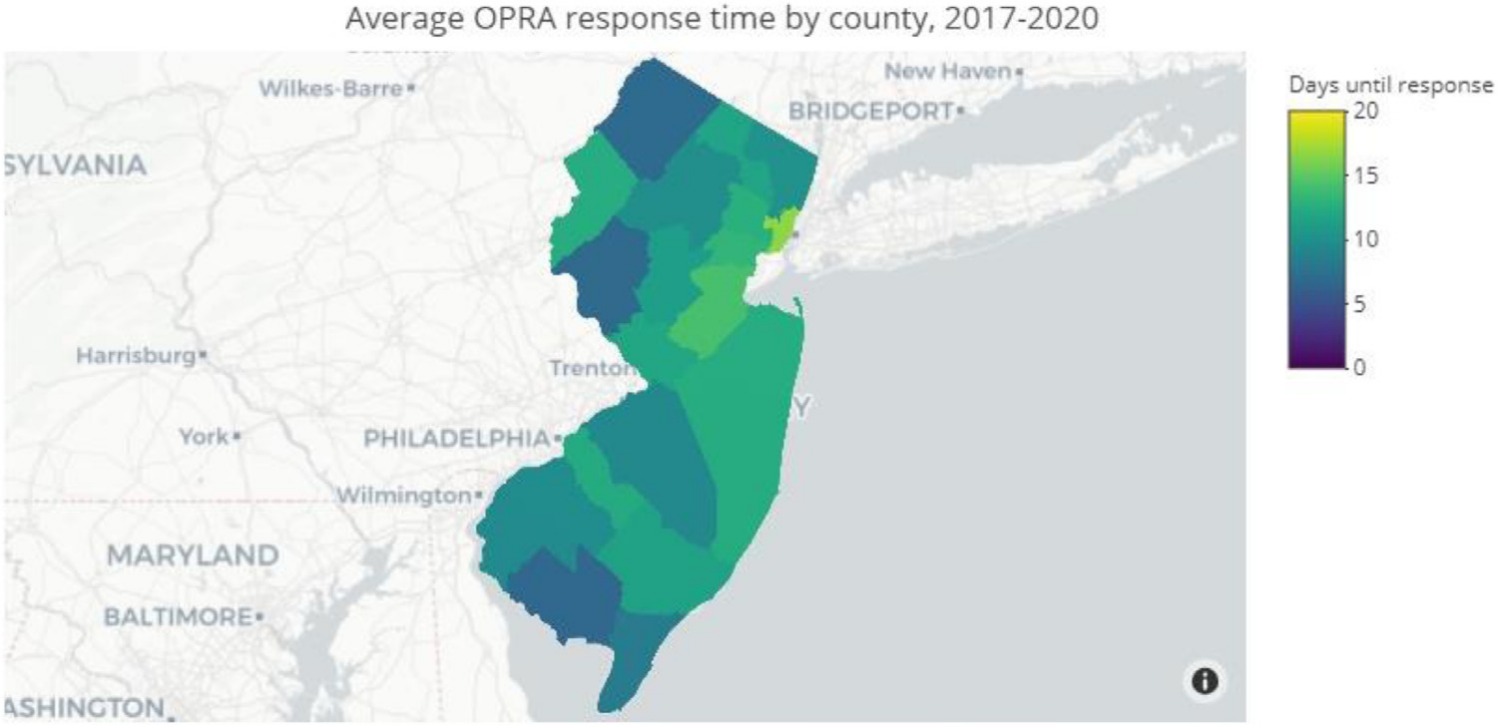
Choropleth map of request response times by county Fig. 2 Choropleth map of average public records request response time in days for New Jersey by county for requests submitted via OPRAmachine.com.

**Fig. 3:**
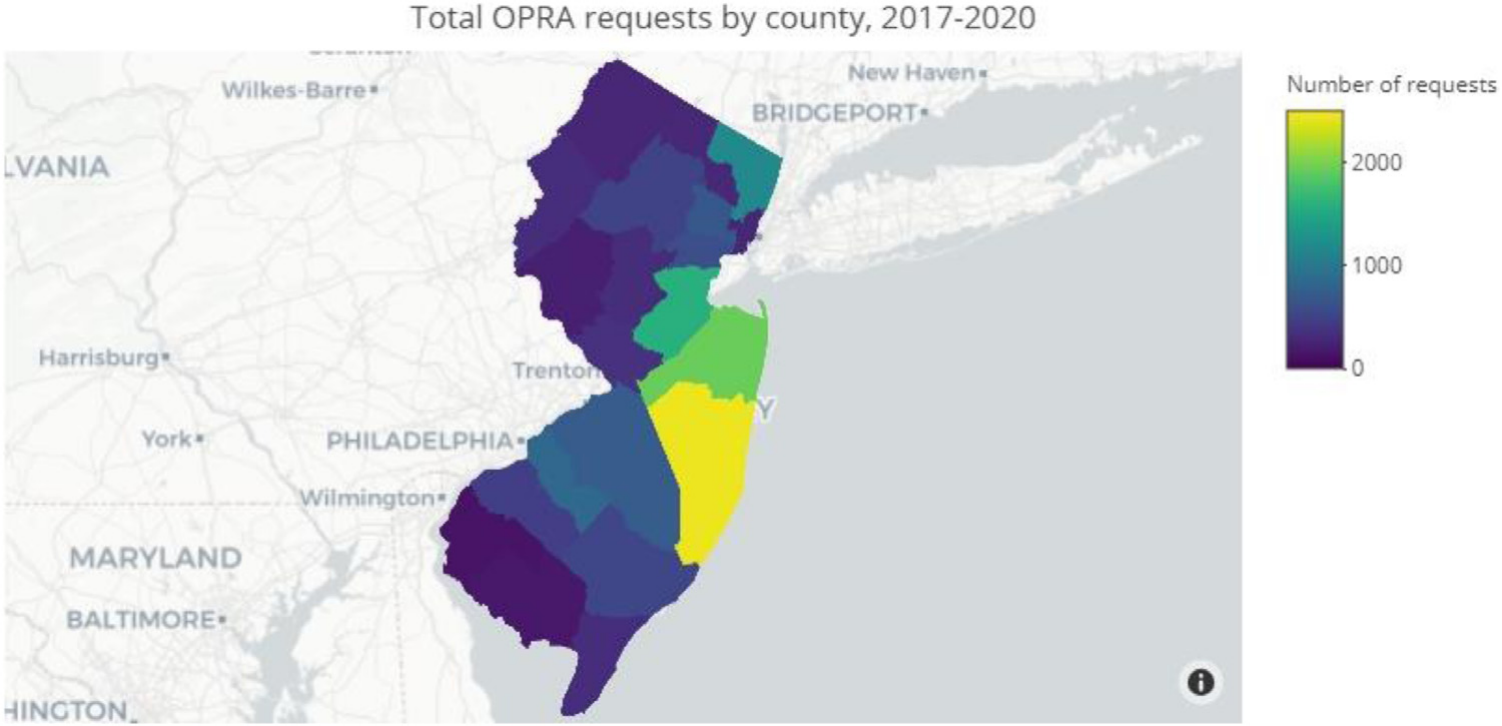
Choropleth map of total requests by county Fig. 3 Choropleth map of number of OPRA requests submitted in New Jersey via OPRAmachine.com by county. Counties were colored based upon the total number of requests submitted to public authorities within each county.

The county-level summary file (“county_summaries.csv”) provides descriptive statistics regarding the number of OPRA requests received by county for each of New Jersey's 21 counties that were represented in the request-level metadata. This file also reports the mean timeframe for responses to requests received from all public authorities located within each county, computed from the data collected for individual requests. Table 2 provides a description of the columns contained within the county-level summary data. Fig. 2 is a choropleth visualization of the average_response_time column joined with a GeoJSON shapefile of New Jersey using the fips_code column for each of the 21 counties represented by these data [6]. The average_response_time value for each county represents the mean difference in time in days between the date the request was created and when a response was last received from the public authority across all requests sent within each county, with the values of tag_string used to map the tag value for each county to the county's corresponding FIPS code in order to create the map. Fig. 3 is a second choropleth and shows the distribution of OPRA requests by county. Both choropleth maps were produced using the Plotly R package [7].

**Table 2:** Description of summarized request data available at the county level.

Column name	Data description	Data type
Name	Proper name of the county	Character
Tag	Tag of the county used to join with requests	Character
fips_code	FIPS code identifying the county	Integer
total_requests	The total number of requests associated with this county	Integer
average_response_time	The mean value of days_until_response for all requests associated with this county	Float
total_requesters	The total number of unique individuals that submitted a public records request to this county	Integer

### 1.3 Public authority tag to proper name mapping

The last CSV file included within the dataset (“authority_tags.csv) provides a mapping between the tags used to describe the public authorities that appear in the request-level data file, which is represented in the tag_string column of the request-level data file. The tag mapping file can be joined with the request data to show the full names of the types of public authorities or geographic locations represented by the tags. Tags were required to be created by the Alaveteli software used for facilitating data collection and can be used to further subset the request data to evaluate trends for specific types of public authorities or locations within New Jersey. A smaller subset of this file was saved as “county_to_tag.csv” to provide a mapping between the county's tag and proper county name, which was used to produce the county-level summaries and related visualizations.

### 1.4 Documents & data released in response to requests

Due to the size of data released in response to all OPRA requests, it was only feasible to publish metadata about the requests for this article, as the size of data released by public authorities and stored on OPRAmachine.com now totals over 50GB in size at the time of publication. This is above the file size limits supported by most data repositories. To access these data, it possible to download released documents by accessing them using the OPRAmachine web interface. This can be done by using the url_title column in the request-level dataset to access the thread of request correspondence via the web interface and download any associated documents or metadata from that location. This can be done like so: https://opramachine.com/request/url_title, where “url_title” is the value of that column for a given request. Similarly, it is also possible to obtain structured data in JSON format for a request by appending “.json” to the URL format described above and additional JSON and RSS endpoints are available for public authorities and custom search queries [8].

## 2 Experimental Design, Materials and Methods

### 2.1 Building the OPRAmachine data collection infrastructure

The author developed the OPRAmachine infrastructure by deploying the open-source Alaveteli software on a Linux virtual private server and configuring it per the software's requirements. The author was motivated to lead the project after evaluating trends in the freedom of information request process and the availability of government information in New Jersey in consultation with journalists, researchers and other frequent requestors of government records. A significant motivation in creating the data collection infrastructure that produced this dataset has been the historic lack of proactive disclosure of public data and documents by government bodies in New Jersey. A 2018 report by the Public Interest Research Group ranked each of the 50 U.S. states based upon the content and ease of use of their state transparency websites with letter grades from A to F. New Jersey has consistently received a “C-“ score or worse [9], necessitating further development of transparency initiatives from both state and private actors. As a state, New Jersey also lacks a dedicated television media market like its larger neighbors in the tri-state area [10], making it even more difficult for citizens to be kept apprised of the activities of government and necessitating increased transparency efforts. By proactively and automatically publishing public records requests and responses through the OPRAmachine platform, this historical lack of transparency can be mitigated through the publication of data collected through public records requests and corresponding metadata showing the temporal and geographic trends in requests.

It should also be noted that the legislative framework of the OPRA law was a key determinant in enabling the collection of data as a part of the OPRAmachine project. The law specifies that requests need not be on a public authority's official request form, merely that they be “written” and invoke the OPRA law in order to be valid [11]. Because OPRAmachine sends requests with the proper language via email to designated email addresses at public authorities, it doesn't utilize the authority's official form, so this part of the OPRA law allows the requests to still be considered legally valid. Other state & local jurisdictions may vary in terms of whether they will accept emailed requests not on the agency's official form, with some states being more restrictive with the manner of submission for public records requests, so that is an additional factor to consider for those who may wish to repeat the data collection process described in this paper and implement a system similar to the system described in this paper.

### 2.2 Records request data collection process

**Fig. 4:**
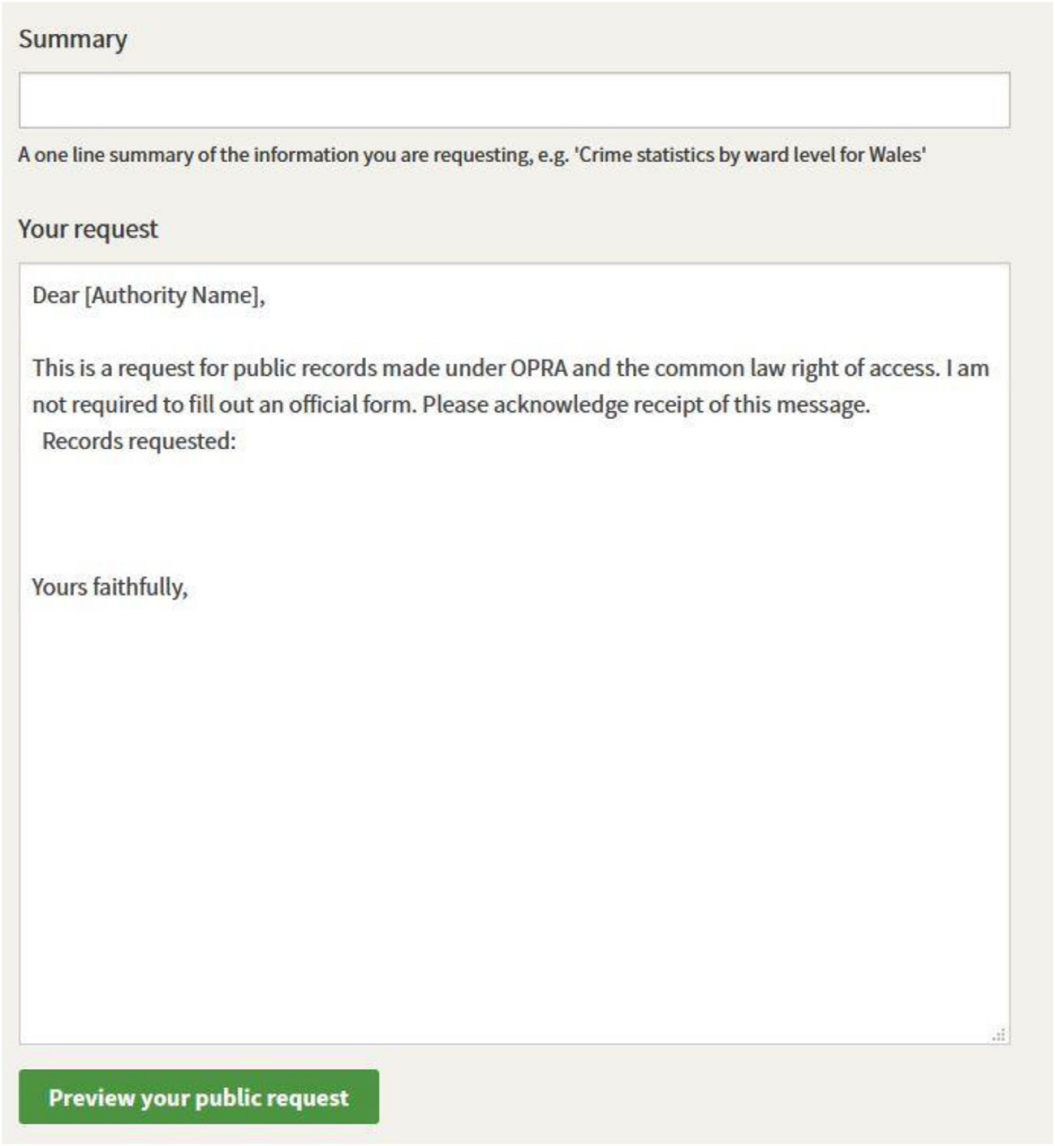
Records request form presented to users Fig. 4 OPRA request form presented to users of OPRAmachine.com during the public records request submission process.

Beginning in October, 2017 and continuing to the present, members of the public were permitted to use a web-based interface to submit public records requests to state, county and municipal public authorities located in New Jersey using the website OPRAmachine.com [5]. Using the state's freedom of information law, the Open Public Records Act (OPRA), individuals completed a brief form describing the information requested from a particular public authority, and the request was sent from the server using a unique email address, with the text of the request, subsequent responses and related metadata stored in a database and retained on the OPRAmachine.com website. The uniquely generated email address contained an identifier that made it possible to track the time between when the request was sent and how long it took to receive a response from the authority in days, which was recorded in the days_until_response column of the request-level data. This approach also allowed any documents & data released in response to the request to be associated with the chain of correspondence between the user and the public authority. During this time period the author facilitated the development of OPRAmachine's data collection infrastructure and ensured that the system continued collecting data. The days_until_response column represents the difference in days between the date a request was initially created and when a response was last received from a public authority. Prior to submitting their records requests, users were required to consent to the publication of the request and data associated with it on the internet. Since the OPRA law allows for the submission of anonymous requests, users were permitted to either submit their requests under their real name, initials, or by using a pseudonym for privacy purposes, such as “Anonymous.” Users were also required to have a valid email address, which was confirmed via an automated email notification, in order to submit a request that was delivered to a public authority. The email addresses of users were only used to confirm that a real person was making the request to prevent spam submissions and were not disclosed to either public authorities or the public. Fig. 4 is a screenshot of the request form on OPRAmachine.com that was presented to users in order to complete their request for public records. The content of the box labelled “Summary” correlates with the values of the title column in the request-level metadata file, while users described their request in detail underneath the “Records requested” portion of the form in this figure.

The web interface used to collect these data utilized Alaveteli, an open-source web application designed for administering freedom of information websites in any jurisdiction created by the UK-based mySociety Foundation [1]. There are over 25 deployments of Alaveteli worldwide, and this particular deployment of the platform was the first to collect data from a single US state rather than an entire country [12]. The software was deployed to a standard Linux server and configured to run the Alaveteli web interface while also acting as a mail server to receive responses to public records requests sent through the unique email addresses created via the website. The interface was customized to fit the requirements of New Jersey's OPRA law, but was otherwise left to the default parameters.

At the time requests were created within the system and sent to the public authority, the Alaveteli software calculated the 7 business deadline for a response to the request, which was recorded in the date_response_required_by column of the OPRA request metadata file. Similarly, the date_very_overdue_after column was created to reflect 20 business days following the submission of the request. This timeline does not represent a legal deadline, but was required to be chosen by the Alaveteli software and can be used as a baseline to determine which requests are long overdue for a response. 20 business days was chosen as the value that represents a request being long overdue because OPRA provides that public authorities should respond to a request within 7 business days, or otherwise as soon as practicable [13].

**Fig. 5:**
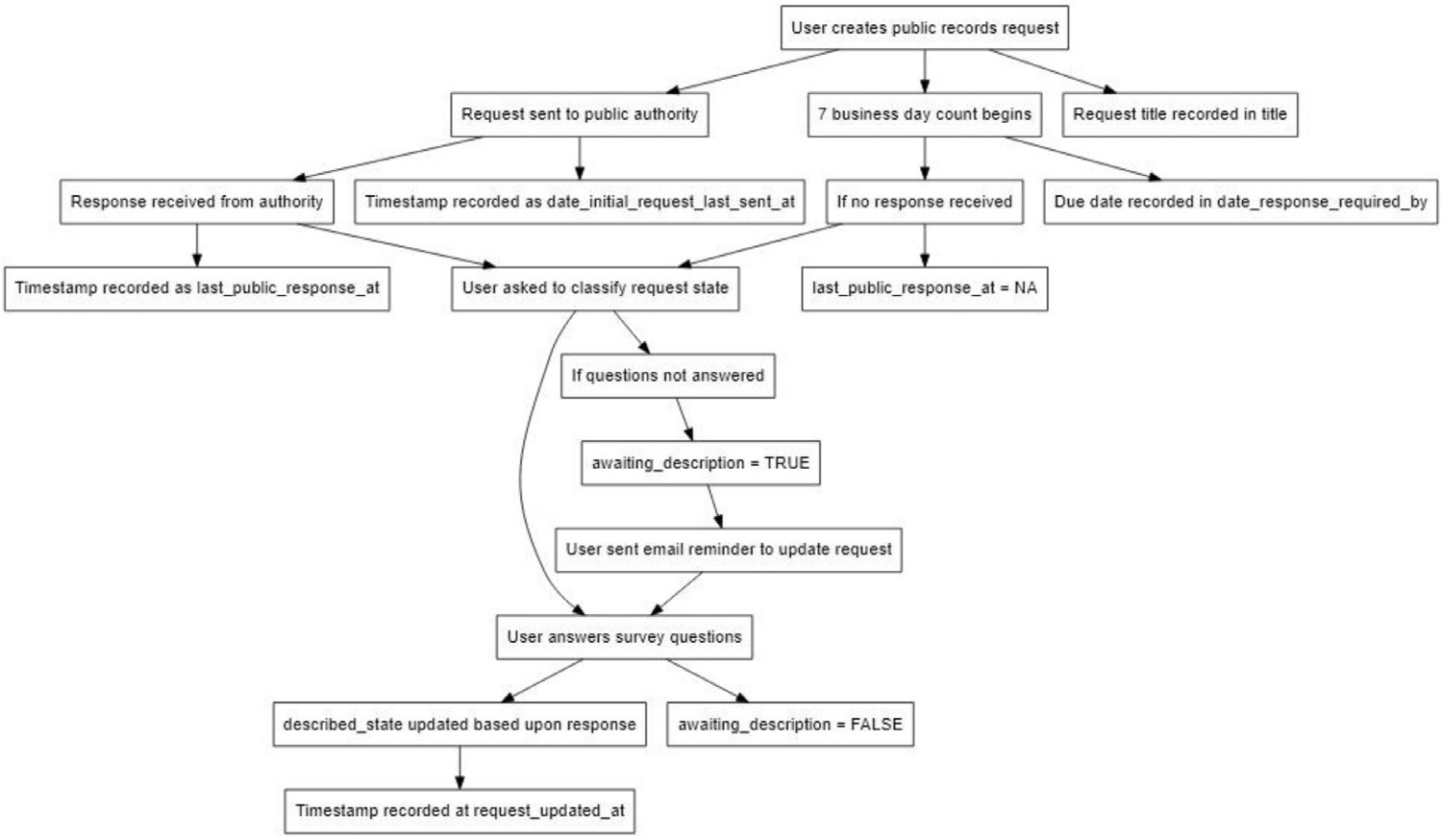
Data collection & public records request submission process Fig. 5 Flowchart of the public records request submission and classification process using Alaveteli on the OPRAmachine.com implementation.

Fig. 5 provides an overview of the data collection process, showing the workflow of how the request metadata was created using this system and how user survey responses are used to classify the request's current state.

Once users received a response to their request from the public authority via an email sent through the platform, the timestamp of the latest response was recorded in the last_public_response_at column in the request metadata and users were subsequently directed to answer a single survey question describing the status of their request. They were asked to check a box next to the survey response that best matched the status of their request. Users were periodically reminded to answer the question about the status of their request via automated email notifications for a period of time after their request received a response if the questionnaire remained unanswered. Each survey choice correlated with a particular request state that was stored in the described_state column based upon the user's response. Users were only permitted to check a single box that they felt best described the status of their request. When the user responded to the questionnaire about their request, the value of the request's awaiting_description column in the request metadata was recorded as FALSE. If users failed to update the state of their request it remained as TRUE until the survey questions were answered for that particular request. The range of possible values for the described_state column were the default states included with the Alaveteli software and no modifications were made to the default request states utilized by Alaveteli.

Table 3 provides a listing of the survey questions that were asked of users that submitted public records requests using the web-based system, and each row shows the values of described_state with which each response corresponds.

**Table 3:** Correlation between survey responses and value of described_state in request-level data file.

Survey response	described_state value
I'm still waiting for my information	awaiting_response
I've been asked to clarify my request	awaiting_clarification
They are going to reply by postal mail	gone_postal
They do not have the information	not_held
I've received some of the information	partially_successful
I've received all the information	successful
My request has been refused	rejected
I've received an error message	error_message

The request metadata was originally stored in a PostgreSQL database table created by Alaveteli. To generate the data files presented in this article, raw tables were exported from the production database server using a COPY SQL statement to store copies of the relevant tables as a CSV file using the psql command-line interface [14]. The exported data was subsequently joined with the names of the requesters and public authorities to create the final data file suitable for publication. The county-level summary data described in Section 1.2 was produced by aggregating the occurrence of requests sent to authorities located within that county using the tag_string column in the request metadata file, as each county was assigned its own tag used to categorize public authorities located within its boundaries. R code for creating the county-level summaries is included as supplemental material (“county_summaries.R”) FIPS codes for each county were joined with the county summary data for each row by county name using the usmap R package [15] and are included in the county summary data to assist in mapping and further geospatial analysis.

## Ethics Statement

While it is commonly understood by requesters that the requests they submit under freedom of information laws, including OPRA, are themselves public records, and courts have held that there is no privacy interest in their contents [16], informed consent was obtained from users of the web interface that their request may be published prior to completing the request submission process.

## Declaration of Competing Interest

The author declares that they have no known competing financial interests or personal relationships which have, or could be perceived to have, influenced the work reported in this article.

## Appendix Supplementary materials

Image, application 1

Image, application 2
